# Stability of SARS-CoV-2 in cold-chain transportation environments and the efficacy of disinfection measures

**DOI:** 10.3389/fcimb.2023.1170505

**Published:** 2023-04-19

**Authors:** Shuyi Peng, Guojie Li, Yuyin Lin, Xiaolan Guo, Hao Xu, Wenxi Qiu, Huijuan Zhu, Jiaying Zheng, Wei Sun, Xiaodong Hu, Guohua Zhang, Bing Li, Janak L. Pathak, Xinhui Bi, Jianwei Dai

**Affiliations:** ^1^GMU-GIBH Joint School of Life Sciences, The Guangdong-Hong Kong-Macau Joint Laboratory for Cell Fate Regulation and Diseases, The State Key Lab of Respiratory Disease, Guangzhou Institute of Respiratory Disease, The First Affiliated Hospital of Guangzhou Medical University, Guangzhou, China; ^2^The Sixth Affiliated Hospital of Guangzhou Medical University, Qingyuan People’s Hospital, Qingyuan, China; ^3^Key Laboratory for Major Obstetric Diseases of Guangdong Province, Guangzhou, China; ^4^Key Laboratory of Reproduction and Genetics of Guangdong Higher Education Institutes, The Third Affiliated Hospital of Guangzhou Medical University, Guangzhou, China; ^5^State Key Laboratory of Organic Geochemistry, Guangdong Provincial Key Laboratory of Environmental Protection and Resources Utilization, Guangzhou Institute of Geochemistry, Chinese Academy of Sciences, Guangzhou, China; ^6^Guangzhou Key Laboratory of Basic and Applied Research of Oral Regenerative Medicine, Guangdong Engineering Research Center of Oral Restoration and Reconstruction, Affiliated Stomatology Hospital of Guangzhou Medical University, Guangzhou, China

**Keywords:** SARS-CoV-2, decay rate analysis, viral activity, low temperature, cold-chain, disinfection method, LED visible light, airflow movement

## Abstract

**Background:**

Low temperature is conducive to the survival of COVID-19. Some studies suggest that cold-chain environment may prolong the survival of severe acute respiratory syndrome coronavirus 2 (SARS-CoV-2) and increase the risk of transmission. However, the effect of cold-chain environmental factors and packaging materials on SARS-CoV-2 stability remains unclear.

**Methods:**

This study aimed to reveal cold-chain environmental factors that preserve the stability of SARS-CoV-2 and further explore effective disinfection measures for SARS-CoV-2 in the cold-chain environment. The decay rate of SARS-CoV-2 pseudovirus in the cold-chain environment, on various types of packaging material surfaces, i.e., polyethylene plastic, stainless steel, Teflon and cardboard, and in frozen seawater was investigated. The influence of visible light (wavelength 450 nm-780 nm) and airflow on the stability of SARS-CoV-2 pseudovirus at -18°C was subsequently assessed.

**Results:**

Experimental data show that SARS-CoV-2 pseudovirus decayed more rapidly on porous cardboard surfaces than on nonporous surfaces, including polyethylene (PE) plastic, stainless steel, and Teflon. Compared with that at 25°C, the decay rate of SARS-CoV-2 pseudovirus was significantly lower at low temperatures. Seawater preserved viral stability both at -18°C and with repeated freeze−thaw cycles compared with that in deionized water. Visible light from light-emitting diode (LED) illumination and airflow at -18°C reduced SARS-CoV-2 pseudovirus stability.

**Conclusion:**

Our studies indicate that temperature and seawater in the cold chain are risk factors for SARS-CoV-2 transmission, and LED visible light irradiation and increased airflow may be used as disinfection measures for SARS-CoV-2 in the cold-chain environment.

## 1 Introduction

Severe acute respiratory syndrome coronavirus 2 (SARS-CoV-2) is the causative agent of coronavirus disease 2019 (COVID-19) and the seventh coronavirus documented to infect humans. The unparalleled COVID-19 pandemic has created a worldwide threat to public health (Koelle et al., 2022). As of February 2023, the total number of confirmed cases of COVID-19 was over 750 million, with more than 6 million deaths globally (https://covid19.who.int/) (2023). Since the emergence of COVID-19, Chinese government employed a series of effective non-pharmaceutical interventions, such as travel restriction, social distancing, extending holidays, and postponing large public events and mass gathering, to contain COVID-19 and prevent more infections. Nevertheless, the resurgence of COVID-19 may be caused by contaminated imported food or packaging *via* cold-chain logistics, where SARS-CoV-2 has been found not only on the surfaces of the frozen food but also on the outer packaging of the frozen food. On August 12, 2020, local authorities in Shenzhen, Guangdong Province, China, detected SARS-CoV-2 nucleic acid on the contaminated surface of frozen chicken wings originating from Brazil, which was the first time SARS-CoV-2 nucleic acid was found on the surface of frozen food in China (Pang et al., 2020; Han et al., 2021). During the recurrence of SARS-CoV-2 in Qingdao, infectious SARS-CoV-2 was isolated from the outer surface of frozen cod, which was the first known case in which infectious SARS-CoV-2 was discovered in a cold-chain environment worldwide (Ma et al., 2021a). A retrospective study found that in the context that there had been no local cases of COVID-19 in Beijing for 56 consecutive days, 98.8% of cases in the next two weeks were linked to the Xinfadi market where a salmon cutting board tested positive for SARS-CoV-2 (Han et al., 2021). According to the virus pedigree analysis, the cases in the new market were caused by the virus strain belonging to B1.1 pedigree s(Yang et al., 2021), which was first identified in Europe and mainly spread in Europe, indicating that the virus strain was transferred from another country. This result suggested that the COVID-19 cases in Beijing at that time might have been caused by imported food. The frequent detection of SARS-CoV-2 on the surface of frozen food or its packaging shows that these events are not coincidental. This finding is a signal that the virus will cause cross-regional COVID-19 transmission through contaminated food. Although the virus titre may be low in actual cold-chain transportation, its infection risk cannot be ignored. Thus, reducing the risk of SARS-CoV-2 transmission in the cold-chain industry has become a challenge. Cold-chain logistics are used for goods such as temperature-sensitive foods and biopharmaceutical products that need to be kept chilled (2°C to 8°C) or frozen (below -18°C) during storage, transportation, and distribution (Li et al., 2021). There have been many studies on the surface viability of SARS-CoV-2 in various frozen meats, fish, and vegetables, which suggest that SARS-CoV-2 can survive on the surface of frozen food (Feng et al., 2021; Bailey et al., 2022). We are however more concerned about the risk of COVID-19 transmission in cold-chain transportation. If the virus contaminates the early packaging or processing process, the continuous low-temperature environment during the storage and transportation of refrigerated food creates conditions conducive to extended virus activity (He et al., 2022). However, studies on the influence of cold-chain environmental factors on the propagation of COVID-19 are lacking.

At present, the method commonly used to disinfect the surface of items is ultraviolet (UV) radiation or sprayed disinfectant containing sodium hypochlorite, peroxyacetic acid, or benzalkonium chlorides. Common disinfectants, including sodium hypochlorite and peroxyacetic acid, can be easily frozen after being sprayed under the cold-chain environment, resulting in a sharp decline in disinfection efficacy (Wu et al., 2022). In addition, a study indicated that the residual disinfectant may also be potentially harmful to personnel in subsequent contact (Choi et al., 2018; Li et al., 2020). Moreover, UV and ionization radiation methods may affect the flavour and texture of goods (Monteiro et al., 2019). These commonly used disinfection methods are thus not suitable for cold-chain transportation. Therefore, it is vital to explore effective methods to inactivate SARS-CoV-2 in a cold-chain environment. Research has shown that visible light can promote virus inactivation (Rathnasinghe et al., 2021). In addition, the research of Jayaweera et al. revealed that airflow is conducive to reduced stability of SARS-CoV-2 (Jayaweera et al., 2020). However, it remains to be determined whether the above two methods are applicable to disinfection during cold-chain transportation.

For study safety, this study was conducted by first constructing a SARS-CoV-2 pseudovirus model with an infection function suitable for environmental factor research. Recent studies have demonstrated that the protruding spike glycoprotein of SARS-CoV-2 interact with the angiotensin-converting enzyme 2 (ACE2) receptor on the membrane surfaces of host cells and mediate virus entry into host cells (Yao et al., 2020; Trougakos et al., 2021). Liu et al. explored the electrostatic adsorption of SARS-CoV-2 from the perspective of interface dynamics by constructing a pseudovirus with the spike structure of SARS-CoV-2 (Liu et al., 2021). Zucker et al. evaluated the efficacy of ozone in disinfection of pseudoviruses on surfaces such as copper and nickel (Zucker et al., 2021), suggesting the feasibility of using pseudoviruses for environmental research. According to the methods reported in the literature (Nie et al., 2020; Yi et al., 2020), we recombined the spike glycoprotein of SARS-CoV-2 onto the surface of a lentivirus similar in shape and size to SARS-CoV-2 and used it as a SARS-CoV-2 pseudovirus model to explore the stability of SARS-CoV-2 in the cold-chain environment. The disinfection effect of physical methods such as light irradiation and airflow on the pseudoviruses was further studied.

## 2 Materials and methods

### 2.1 Production of SARS-CoV-2 pseudovirus

SARS-CoV-2 pseudovirus with spike glycoprotein was constructed as previously described (Neerukonda et al., 2021). The full-length open reading frame of the spike gene of the SARS-CoV-2 isolated from Wuhan hu-1 (Genbank registration number: YP_009724390.1) was synthesized by Genewiz (Genewiz Biotech Co., Ltd., Suzhou, China) and cloned the synthesized fragment into pcDNA3.1(+) vector (Shenggong Biological Engineering Co., Ltd., Shanghai, China) to construct pcDNA3.1-SARS-CoV-2-S plasmid. Then pseudovirus bearing the spike glycoprotein and carrying the EGFP reporter gene were produced in HEK293T cells. Briefly, HEK293T cells grown to 80% confluency in the dishes were co-transfected with the pLV-EGFP-N plasmid, psPAX2 expression plasmid, and pcDNA3.1-SARS-CoV-2-S plasmid using polyethyleneimine (PEI, Polysciences, USA). Meanwhile, lentiviruses used as the control group were packaged under the same conditions with pLV-EGFP-N, psPAX2, and pMD2.G plasmids. Pseudovirus supernatants were harvested and purified by ultrahigh-speed centrifuges with space 20000 g/min (XPN-100, Beckman, USA) approximately 72 h post-transfection. The pseudovirus was uniformly adjusted to 6.0 ± 0.2 log_10_TU/mL by ultrapure water or PBS after titer determination, then packed into multiple tubes and stored at -80°C until use. All frozen viruses were used only once to avoid inconsistencies that could have resulted from repeated freezing-thawing cycles. Pseudoviruses will be used to explore the impact of the cold chain environment on viruses, and to explore the ability of light and other intervention methods to align inactivation experiments.

### 2.2 Titration of SARS-CoV-2 pseudovirus

To determine the titer of the virus, 293T-hACE-2 cells which are generously gifted by Weisheng Guo (Guangzhou Medical University) were seeded in a 96-well plate and incubated for 12 h, then added serially diluted SARS-CoV-2 pseudovirus supernatant in DMEM. After 72 h incubation, the cells were imaged under fluorescent microscopy and the presence of viral particles was determined by observing green fluorescence. We refer to the method of Barczak et al. for virus titer determination (Barczak et al., 2015), which reduces false negative results effectively. Specifically, the genomes of 293T-hACE-2 cells that were infected with SARS-CoV-2 pseudovirus were extracted using TIANamp Genomic DNA Kit (Tiangen Biotech Co., Ltd., Beijing, China) according to the manufacturer’s instruction. The viral titer was estimated by quantification of viral copy number in infected cells through the amplification of SARS-CoV-2 pseudovirus-specific transgene (Long terminal repeat, LTR) and a single copy reference gene (Albumin, ALB) using real-time quantitative PCR assay (SYBR^®^ Green Premix Pro Taq HS qPCR Kit (Accurate Biology Co. Ltd., Changsha, China) on a Light Cycler 96 PCR machine (Bio-rad laboratories, California, USA) according to the protocol of previously report (Barczak et al., 2015). Virus titer = (copy number of LTR/2)/copy number of ALB)*2*number of transduced cells*virus dilution ratio/volume of the used virus. The 2-fold factor reflects the presence of two alleles of the albumin gene. The plasmid used for virus packaging will make the virus genome carry two copies of the LTR gene, and ALB albumin is a single-copy gene carried by cells. The sequences of ALB and LTR were amplified by real-time quantitative PCR using the following primers: ABL-forward: 5´-TTTGCAGATGTCAGTGAAAGAGA-3´; ABL-reverse: 5´-TGGGGAGGCTATAGAAAATAAGG-3´; LTR-forward: 5´- CTAGCTCACTCCCAACGAAGA-3´; LTR-reverse: 5´- GGTCTGAGGGATCTCTAGTT-3´; The copy number and titer of SARS-CoV-2 pseudovirus per cell was calculated as the previously report (Barczak et al., 2015).

### 2.3 Identification of virus morphology and infectivity

Western blotting assay was used to identify of spike glycoprotein in SARS-CoV-2 pseudovirus. Further, the fluorescence of cells infected by pseudovirus was detected by fluorescence inversion microscope to determine whether pseudovirus can infect cells. The same titer of pseudovirus and lentivirus were mixed with 6× SDS-sample buffer and the mixture was heated for 5 min at 100 °C. In addition, the total cell protein of cells infected by pseudovirus or lentivirus was collected for western blotting assay. The methods of western blotting assay refer to the previous study (Guo et al., 2022). The membrane was incubated with SARS-CoV-2 spike RBD antibody(1:1000, #69323, Cell Signaling Technology, USA), Human Immunodeficiency Virus type 1 (HIV-1) Gag-p24 antibody(1:1000, 11695-RP02, Sino Biological, Shanghai, China) and subsequently incubated with horseradish peroxidase (HRP)-conjugated secondary antibodies (Goat Anti-Rabbit IgG, 1:2000, SA00001-2, Proteintech; Goat Anti-mouse IgG, 1:2000, SA00001-1, Proteintech, USA). The bands were visualized with ECL reagents (A38555, Thermo Fisher Scientific, USA) using Image Quant LAS 4000 system (GE Healthcare, Waukesha, USA), and quantified using Image J V1.8.0 software.

In order to detect whether spike glycoprotein is expressed on the surface of pseudovirus, we used transmission electron microscopy (TEM) to observe whether pseudovirus has a spike structure, with lentivirus as the control group. The virus was resuspended in ultrapure water containing 0.005% Bovine serum albumin (Sigma-Aldrich, USA). Purified and filtrated SARS-CoV-2 pseudovirus was firstly fixed in 2% glutaraldehyde (Leagene, Beijing, China), and then was negative-stained through 1% Phosphotungstic acid (Solarbio, Beijing, China). Finally, the SARS-CoV-2 pseudovirus samples were collected on a copper grid (Zhongjingkeyi, Beijing, China). The samples were observed using a transmission electron microscope (H-7650, Hitachi, Japan).

### 2.4 Decay rate calculations

Numerous studies have shown that once many microorganisms, including respiratory pathogens, leave their original environment, the change in relative survival rate C_t_/C_0_ with time usually follows an exponential function (Dunklin and Puck, 1948; Ahmed et al., 2020; Guo et al., 2021).


(1) $${\text{C}}_{\text{t}}{\text{/C}}_{0}=e^{-kt}$$

According to equation (1)


(2) $$\text{Ln }\left({\text{C}}_{\text{t}}{\text{/C}}_{0}\right)\;=-kt$$

Ln (C_t_/C_0_) has a linear relationship with time *t*. Then the virus decay rate constant *k* can be calculated as the regression slope of Ln (C_t_/C_0_) versus time using linear least squares regression. In this formulation, C_t_ was the virus concentration at time *t*, and C_0_ was the initial virus concentration at the beginning of the experiment (i.e., *t*=0). T was the duration of the SARS-CoV-2 under different conditions, and *k* was called the decay rate of the SARS-CoV-2 under different conditions. In the linear regression analyses, the observed viable titer of SARS-CoV-2 pseudovirus concentrations was linearized using the natural log (ln)-transformation of the normalized concentrations as shown in equation (2)

### 2.5 Stability of SARS-CoV-2 pseudovirus under different surfaces

Pan et al. reported that the viral loads in the throat swab and sputum sample had peaked at 5~6 days after symptom onset, at approximately around 10^4^ to 10^7^ copies per milliliter during this time (Pan et al., 2020). The mean concentrations of SARS-CoV-2 pseudovirus in the studied at day 0 ranged from 6.0 ± 0.2 log_10_TU/mL. The SARS-CoV-2 pseudovirus has been suspended in DMEM containing the 2% (vol/vol) fetal bovine serum. The 50 μL of SARS-CoV-2 pseudovirus (C_0_) was placed on the four types of common surfaces, including Teflon, cardboard, stainless steel, and PE plastic, for 3 days at 16°C and 70% relative humidity, which is similar to the outbreak of COVID-19 in Guangzhou. The Teflon film had been used as a positive control of smooth material due to the low coefficient of friction characteristic. After the equal amount of SARS-CoV-2 pseudoviruses was placed on the surface of different media, rinsed 10 times with 200 μL DMEM containing the 2% (vol/vol) fetal bovine serum to recover the pseudoviruses. The recovered pseudoviruses immediately infects the 293T-hACE2 cells to quantify the titer (C_t_) of pseudovirus as described above. Then, decay curves over time and decay rate constants *k* of SARS-CoV-2 pseudovirus were analyzed as previously reported (Ahmed et al., 2020).

### 2.6 Stability of SARS-CoV-2 pseudovirus under cold-chain temperature and in seawater at -18°C and repeated freeze-thawing cycles

The mean concentrations of SARS-CoV-2 pseudovirus in the studied at Day 0 ranged from 6.0 ± 0.2 log_10_TU/mL. The SARS-CoV-2 pseudovirus has been suspended in DMEM containing the 2% (vol/vol) fetal bovine serum. Seawater in the South China Sea near Hainan Province, which is rich in seafood resources is collected for experimental research. The 50 μL of SARS-CoV-2 pseudovirus (C_0_) was suspended in seawater, then placed under 25°C, 4°C, 0°C, -18°C, and -70°C for 20 days. Meanwhile, in order to eliminate the influence of salt ions in seawater, we use deionized water as the control group. The virus solutions were deposited at -18°C on the surface of the PE plastic respectively to mimic cold chain transport. PE plastic, which is a common food packaging material in actual cold chain transportation had been used to simulate the outer packaging of frozen products. The virus suspension is subjected to multiple freeze-thaw cycles within the temperature range of -18 °C~25 °C, simulate the effects of freeze-thaw on the SARS-CoV-2 on the outer packages and investigate the effect of remaining salt ions on the SARS-CoV-2. SARS-CoV-2 pseudoviruses were recovered using 200μL DMEM containing the 2% (vol/vol) fetal bovine serum. Infect 293T-hACE2 cells with the recovered pseudovirus for 72 hours, and extract the cell genomic DNA. Subsequently, the pseudovirus content, the decay curves over time, and the decay rate constants *k* of the virus were analyzed as the method mentioned above.

### 2.7 The disinfection potential of LED visible light illumination and airflow movement on SARS-CoV-2 pseudovirus under cold-chain conditions

50μL of SARS-CoV-2 pseudovirus (C_0_) which titer is 6.0 ± 0.2 log_10_TU/mL was placed at -18°C condition on the surface of packaging materials commonly used for marine products, including PE plastic and corrugated carton. The standard LED visible light contains spectral wavelengths of 450 nm to 780 nm with a luminous intensity of 4 mW/cm^2^. The SARS-CoV-2 pseudovirus had been exposed under visible light for 180 min. The virus groups treated with darkness were used as negative controls. The SARS-CoV-2 pseudovirus had been exposed under the airflow movement with an airflow of 3 m/s at -18°C for 32h. The airflow is generated by a fan with adjustable wind speed added in the refrigerator, and the virus is placed at the position where the airflow speed is 3m/s. The virus groups treated with calm were used as negative controls. SARS-CoV-2 pseudoviruses were recovered using 200 μL DMEM containing the 2% (vol/vol) fetal bovine serum and the recovery was repeated three times and cultured with 293T-hACE2 cells to quantify the titer (C_t_) as described above. Subsequently, the decay curves over time and the decay rate constants *k* of the virus were analyzed as described above.

### 2.8 SARS-CoV-2 pseudovirus RNA damage assay

As mentioned above, the titer of SARS-CoV-2 pseudovirus has been adjusted to 6.0 ± 0.2 log_10_ TU/mL before cryopreservation. After thawing, add 50 μL pseudovirus suspension on the PE plastic surface respectively, and then place them at -18 °C for LED light or no light treatment for 10 minutes. At the same time, the pseudovirus without any treatment after thawing was used as the control group. The total RNA of each groups SARS-CoV-2 pseudovirus was extracted using Steady Pure Virus DNA/RNA Extraction Kit (Accurate Biology Co. Ltd., Changsha, China), and further evaluated RNA damage using agarose gel electrophoresis. 1× TAE was used to prepare 1.5% agarose gel. 16μL pseudovirus RNA is mixed with 4μL RNA loading buffer, and then added to agarose gel for electrophoresis separation at 100V for 30min. All tools and reagents used in agarose gel electrophoresis need to remove RNase. The gray-scale scanning analysis of pseudoviruses genome RNA degradation was performed using Image J software (Java-based image-processing and analysis software, National Institutes of Health, USA).

### 2.9 Statistical analysis

All the data are presented as the means ± standard deviation (SD) from three independent experiments. All statistical analyses were performed using GraphPad Prism 8.3.1 (GraphPad Software, La Jolla, CA, USA). Student’s t-test was used to determine the differences between the two groups. *p* values < 0.05 (*p* < 0.05) were considered to indicate a significant difference. The first-order decay rate constant of pseudovirus at each point time was calculated by linear regression using GraphPad Prism Version 8.3.1 (GraphPad Software, La Jolla, CA, USA). As a comparison metric, *r*^2^ was used to evaluate the proportion of the variance explained by the linear model.

## 3 Results

### 3.1 Construction and properties of SARS-CoV-2 pseudovirus

**Figure 1:**
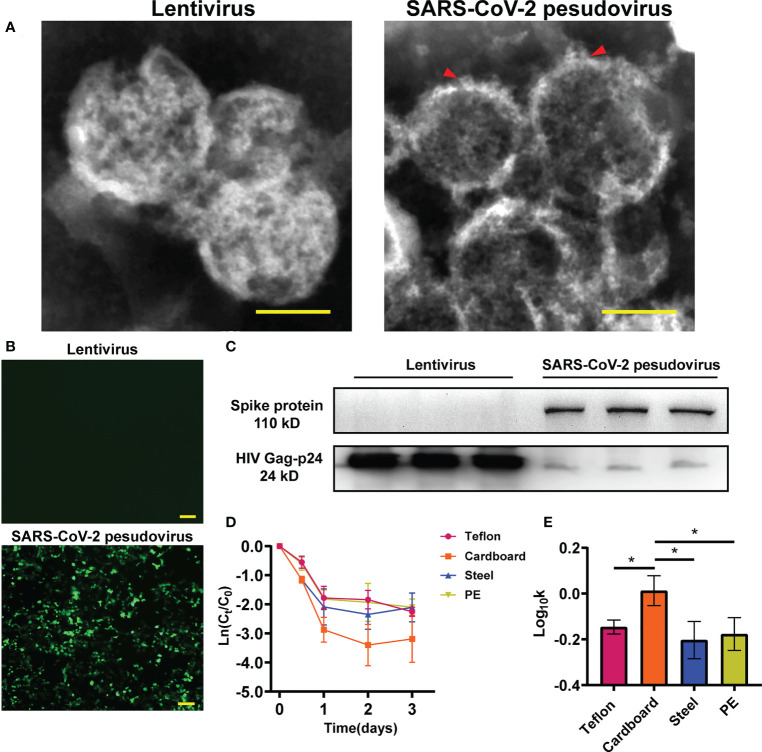
Identification of SARS-CoV-2 pseudovirus. **(A)** Structure analysis of lentivirus and SARS-CoV-2 pseudovirus using Transmission electron microscopy (TEM) assay. **(B)** Infectivity of SARS-CoV-2 pseudovirus on 293T-hACE-2 cells. **(C)** The protein expression of S-spike in lentivirus and SARS-CoV-2 pseudovirus, HIV Gag-p24 is a lentivirus capsid protein and also exists in pseudovirus. **(D)** Decay curves of SARS-CoV-2 pseudovirus over 3 days on the surface of Teflon film, cardboard, stainless steel, and PE plastic. The measurements were linearized premised on first-order decay, in which the natural log (ln)-transformed measured concentration at each time point was divided by the concentration at time zero. **(E)** Log10-transformed mean pseudovirus decay rate constants k of SARS-CoV-2 pseudovirus on the surfaces of different materials at 16°C. Scale bar: 100 nm **(A)** and 200 μm **(B)**. *p<0.05.

To investigate the infectivity and stability of SARS-CoV-2 in a cold-chain environment, SARS-CoV-2 pseudovirus particles were constructed using a lentivirus packaging system. Transmission electron microscopy (TEM) was performed on harvested viral supernatant to visually confirm the production and assembly of SARS-CoV-2 pseudovirus. TEM images of negatively stained SARS-CoV-2 isolated from patients previously have revealed that SARS-CoV-2 particles are polyhedral spheres 60-140 nm in diameter. Distinctive spikes protrude from the outer membrane of virus particles, affording virions a solar corona appearance (Mittal et al., 2020; Zhu et al., 2020). In this study, the particle size and structure of SARS-CoV-2 pseudovirus were similar to those of wild-type virus particles observed by Zhu et al. (**Figure 1A**). Our results showed that pseudoviruses and lentiviruses were spherical enveloped viruses with diameters of 100 nm~120 nm. In particular, the spike structure on the surface of the pseudovirus indicates that the spike glycoprotein is assembled on the surface of the pseudovirus rather than inside. Numerous studies have confirmed that SARS-CoV-2 infects human cells by binding to the cell surface protein ACE2 through the receptor-binding domain (RBD) of its spike (S) glycoprotein (Peng et al., 2021; Trougakos et al., 2021; Jackson et al., 2022). The pseudovirus we constructed has been confirmed to have a functional spike glycoprotein that can recognize the cell membrane. SARS-CoV-2 pseudovirus showed much higher infectivity in 293T-hACE-2 cells than in regular HEK293T cells (**Figure 1B**). To further confirm that the spike glycoprotein of SARS-CoV-2 was inserted into the pseudovirus model, we detected the spike glycoprotein in the total protein of lentivirus and SARS-CoV-2 pseudovirus by Western blotting (**Figure 1C**).

To determine the ability of the SARS-CoV-2 pseudovirus to mimic SARS-CoV-2, we investigated the stability of SARS-CoV-2 pseudovirus on different surfaces including cardboard, polyethylene (PE) plastic, stainless steel, and Teflon film at 16°C and 70% relative humidity (RH). The decay of SARS-CoV-2 pseudovirus on the surfaces was analysed using linear regression. The declining concentration and the mean decay rate constants *k* for SARS-CoV-2 pseudovirus on the different surfaces are shown in **Figures 1D**, **E**. The SARS-CoV-2 pseudovirus was stable on PE plastic, stainless steel, and Teflon and remained viable for three days on these surfaces. The mean decay rate revealed that the survival time of SARS-CoV-2 pseudovirus was not obviously different on the nonporous surfaces at 16°C; however, the survival time was surprisingly less on porous surfaces such as cardboard than on nonporous surfaces. Regarding the infectivity of SARS-CoV-2 pseudovirus on various surfaces, the mean decay rate constants *k* ranged from 0.67/day on PE plastic to 1.04/day on cardboard, with *r*^2^ values ranging from 0.65 to 0.67 (**Table S1**). The results indicate that SARS-CoV-2 pseudovirus remains stable for a longer period on nonporous surfaces.

### 3.2 The decay of SARS-CoV-2 pseudovirus in a cold-chain environment

**Figure 2:**
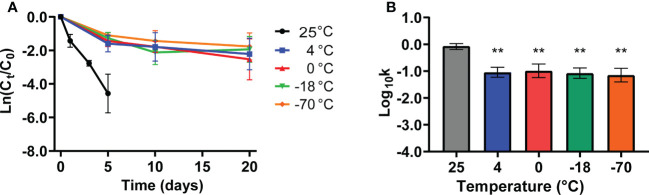
The stability of SARS-CoV-2 pseudovirus under cold-chain temperatures. **(A)** The decay curves of SARS-CoV-2 pseudovirus on PE plastic over 20 days under different temperatures. **(B)** The decay rate constants k of SARS-CoV-2 pseudovirus on PE plastic under different temperatures. **p <0.01 compared with 25°C.

Cold-storage conditions are conducive to maintaining virus vitality. Hence, we evaluated the stability of SARS-CoV-2 pseudovirus under different refrigeration temperatures such as 4°C, 0°C, -18°C, and -70°C on the PE surface for 20 days. The decay of viable SARS-CoV-2 pseudovirus occurred in a temperature-dependent manner, and the declining concentration of SARS-CoV-2 pseudovirus at room temperature was higher than that at other cold-chain temperatures; no SARS-CoV-2 pseudovirus was detected at room temperature on the sixth day (**Figure 2**). In addition, SARS-CoV-2 pseudovirus could survive for over 20 days under cold-chain temperatures (**Figure 2A**). Accordingly, the decay rate constants *k* of SARS-CoV-2 pseudovirus under different cold-chain conditions were significantly lower than those at room temperature (**Figure 2B**). Regarding the stability of SARS-CoV-2 at various temperatures, the mean decay rate constants *k* ranged from 0.08/day at -70°C to 0.87/day at room temperature, with *r*^2^ values ranging from 0.57 to 0.91, respectively (**Table S2**). These results indicated that cold-chain temperature conditions, compared to room temperature conditions, could decrease the decay rate of SARS-CoV-2 pseudovirus and increase the risk of SARS-CoV-2 transmission.

**Figure 3:**
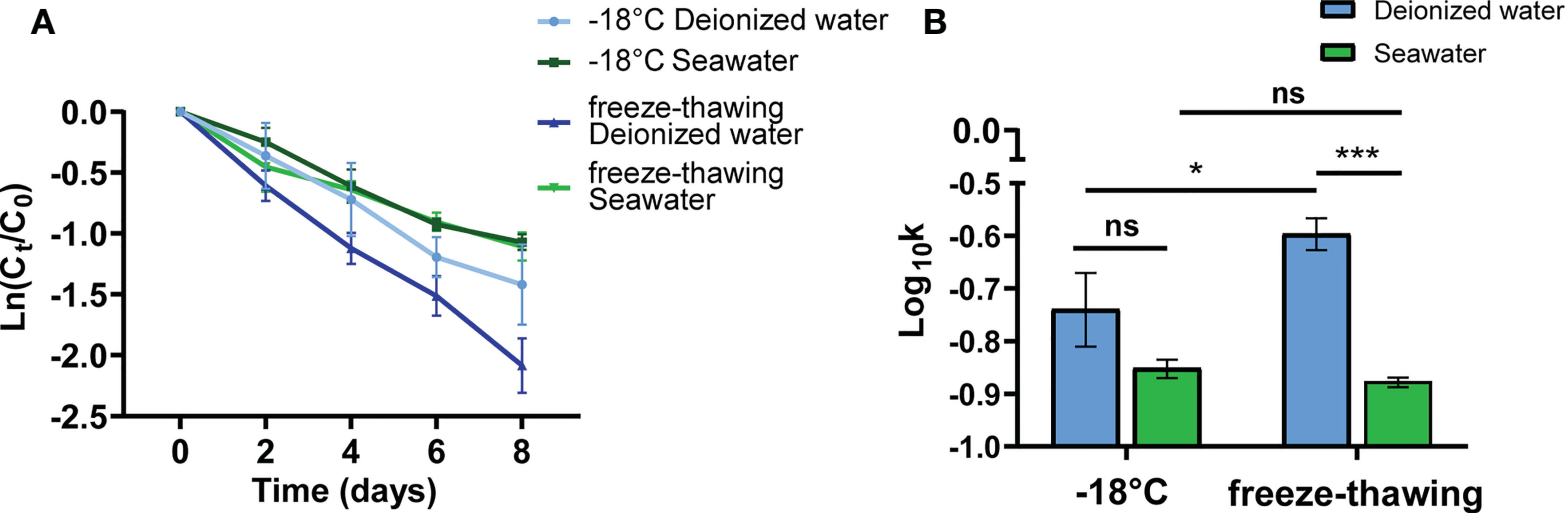
The protective effect of seawater on SARS-CoV-2 pseudovirus at -18°C. **(A)** The decay curves over time (days) of SARS-CoV-2 pseudovirus, which was solutes in seawater or deionized water, on PE plastic at -18°C or under freeze-thawing. The treatment condition is freezing and thawing once a day, and virus activity is tested once every two days. **(B)** Decay rate constants k of SARS-CoV-2 pseudovirus at -18°C and under the condition of freeze-thawing cycles. ns., no significantly difference; *p<0.05; ***p<0.001.

The reported cases of exogenous SARS-CoV-2 infection in seafood markets in different cities indicate that the presence of seawater may be beneficial to the survival of SARS-CoV-2. As SARS-CoV-2 RNA has been frequently detected on the outer packages of frozen seafood, we further investigated the potential protective effect of seawater on SARS-CoV-2. The SARS-CoV-2 pseudovirus was suspended in seawater and deionized water. The freeze−thaw group was subjected to repeated freeze−thaw cycles, and continuous freezing at -18°C was used as the control condition. The purpose of this experiment was to simulate the freezing and thawing caused by the short time of contact with warm air during the loading and unloading of cold-chain goods. Interestingly, the results showed that the decreased range of SARS-CoV-2 pseudovirus activity in deionized water was higher than that in seawater under -18°C and repeated freeze−thaw conditions (**Figure 3A**). However, there was no significant difference in the decay rate constants *k* of SARS-CoV-2 pseudovirus between deionized water and seawater at -18°C continuous freezing. Compared with that of the deionized water group, the activity of SARS-CoV-2 pseudovirus was attenuated slowly during repeated freeze-thaw cycles (**Figure 3B**). Compared with the virus attenuation rate of the four groups, the virus attenuation rate of the deionized water freeze−thaw group was the highest (0.25 ± 0.01/d), followed by that of the deionized water -18 °C group (0.18 ± 0.02/d), and the virus attenuation rate of the seawater resuspension group was lower than that of the deionized water groups (**Table S3**). The above results show that compared with deionized water, seawater effectively maintained SARS-CoV-2 pseudovirus survival under the condition of repeated freezing and thawing, suggesting that the seawater in frozen cargo may increase the risk of SARS-CoV-2 transmission.

### 3.3 The disinfection potential of LED visible light illumination on SARS-CoV-2 pseudovirus under cold-chain conditions

**Figure 4:**
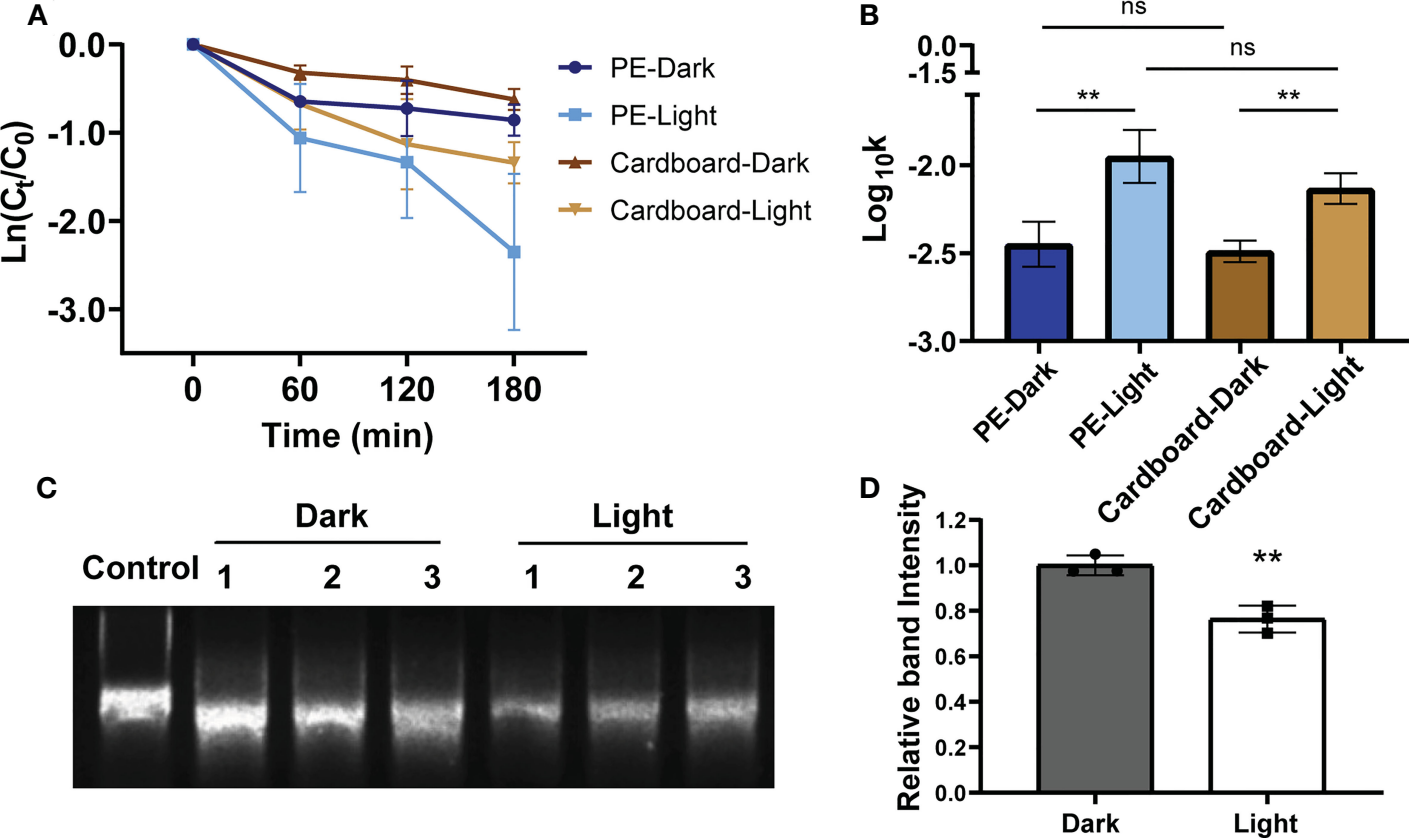
The disinfection potential of LED visible light from 450 nm to 780 nm on SARS-CoV-2 pseudovirus at -18°C. The decay curves over time (min) of SARS-CoV-2 pseudovirus on PE plastic **(A)** and cardboard at -18°C that expose or un-expose to LED visible light (450 nm ~ 780 nm). The decay rate constants k of LED visible light exposed SARS-CoV-2 pseudovirus on the PE plastic and cardboard **(B)** at -18°C were significantly higher than that of light non-exposed control. **(C)** Genomic RNAs of SARS-CoV-2 pseudovirus on the PE plastic that was exposed or unexposed to LED visible light at -18°C were determined by agarose gel electrophoresis, control: SARS-CoV-2 pseudovirus RNA without any treatment **(D)** The RNA band intensities were quantified analysis using gray-scale scanning software. ns, no significantly difference; **p<0.01.7.

Previous studies have shown that sunlight can inactivate viruses(Hadi et al., 2020; Ratnesar-Shumate et al., 2020; Sagripanti and Lytle, 2020), suggesting that we can use visible light irradiation in the cold-chain transportation process. To explore the possible methods to inactivate SARS-CoV-2 under the cold-chain environment, SARS-CoV-2 pseudovirus-contaminated surfaces that are usually used in seafood packing materials, including PE plastic and corrugated cardboard, were exposed to LED visible light of spectral wavelengths from 450 nm to 780 nm at -18°C for the indicated time. The survival of SARS-CoV-2 on the PE plastic and cardboard declined during exposure to LED visible light compared with that of the no-light-treatment controls (**Figure 4A**). In addition, a 2-fold reduction in virus load was observed at 180 min on PE plastic and cardboard in the presence of LED visible light compared with that of the controls. Furthermore, the decay rate constants *k* showed that irradiation with LED visible light significantly increased the decay rate of SARS-CoV-2 pseudovirus on PE plastic and cardboard at -18°C compared with that of the no-light-exposure controls (**Figure 4B**). Regarding the infectivity of SARS-CoV-2 pseudovirus upon LED visible light treatment, the mean decay rate constants *k* ranged from 0.004/min on PE plastic to 0.007/min on cardboard surfaces. However, in the dark condition, the mean decay rate constants *k* ranged from 0.002/min on PE plastic to 0.003/min on the cardboard surface (**Table S4**). Some studies have shown that solar radiation damages viral RNA(Sagripanti and Lytle, 2020). To preliminarily explore the mechanism by which visible light affects the survival of viruses, we conducted LED light irradiation of viral RNA and tested the viral RNA integrity through agarose gel electrophoresis. The electrophoresis band of LED visible light-treated pseudovirus RNA was lighter than that of the no-light-treatment controls (**Figure 4C**). Quantitative analysis of RNA bands further showed that LED visible light treatment significantly decreased the total RNA of pseudovirus compared with that of the no-light-treatment group RNA (**Figure 4D**). These results suggest that LED visible light can be used as a simple and effective cryogenic physical disinfection tool, which can be applied to reduce the risk of virus carriage in a cold-chain environment.

### 3.4 The effect of airflow on the viability of SARS-CoV-2 pseudovirus in a cold-chain environment

**Figure 5:**
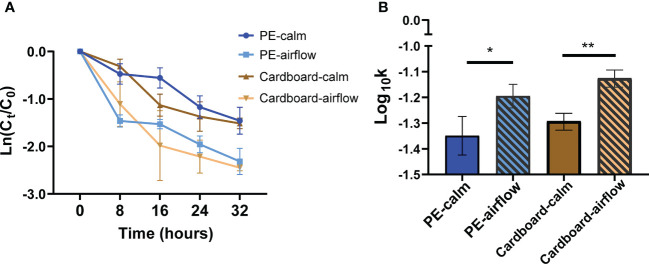
The effect of airflow movement on the viability of SARS-CoV-2 pseudovirus at -18°C. The decay curves over 32 hours of SARS-CoV-2 pseudovirus on PE plastic and cardboard **(A)** at -18°C that was treated or untreated with the airflow movement (3 m/s). The decay rate constants k of wind-treated SARS-CoV-2 pseudovirus on the PE plastic **(B)** and cardboard at -18°C were significantly higher than that of non-airflow movement controls. *p<0.05; **p<0.01.

At present, the low-temperature transportation conditions of food are often set according to different food types. The wind speed at the cooling air outlet in most cold storage is greater than 6 m/s, while the wind speed at the place where the items are stored in the cold storage space is within the range of 0.5-4 m/s, which is quite different in specific situations (Bishnoi and Aharwal, 2021; McSharry et al., 2021). We preliminarily set a wind speed of 3 m/s as the treatment condition and then further analysed the effect of airflow on the viability of SARS-CoV-2 pseudovirus at -18°C. The decay curve over time of SARS-CoV-2 pseudovirus on PE plastic and cardboard at -18°C showed a decrease in survival when pseudoviruses were exposed to airflow compared with that in a nonairflow environment (**Figure 5A**). The decay rate constants *k* of SARS-CoV-2 pseudovirus on PE plastic and cardboard at -18°C were markedly increased by 1.5-fold in the airflow group compared with the nonairflow group (**Figure 5B**). By comparing the average decay rate of the above four treatment groups (**Table S5**), we found that the attenuation rate of pseudoviruses in the airflow group, whether on cardboard or PE plastic, was faster than that in the static group. The above results indicate that increased airflow may promote the inactivation of SARS-CoV-2 during cold-chain transportation.

## 4 Discussion

Since SARS-CoV-2 is categorized as a biosafety level 3 (BSL3) agent, it is difficult to conduct research in many conventional biological laboratories. To simulate the survival of virus particles in different environments, we must use a virus that is similar to SARS-CoV-2 in morphology and infectivity to reduce harm to the human body. In research on virus infection mechanisms and vaccine development, pseudoviruses can simulate the structure and infectivity of the original virus to a certain extent. At present, according to the application needs, scholars use different virus models as pseudoviruses of SARS-CoV-2. For example, virus structural proteins are used to create virus-like particles (VLPs) for vaccine development and research (Naskalska et al., 2018). The purpose of this study was to explore the impact of environmental factors on the survival of viruses. A pseudovirus model with an infectious ability and appearance similar to the real virus is needed. However, VLPs have only the structure of viruses and no infective activity, which cannot meet the needs of this study. In addition, the commonly used method to construct pseudoviruses is to recombine different structural components of the virus into HIV (human immunodeficiency virus) or VSV (vesicular stomatitis virus) to build models. To simulate the shape of SARS-CoV-2 as much as possible, we used the spherical recombinant HIV virus instead of the bullet-type VSV virus (Chen and Zhang, 2021). Pseudoviruses offer great advantages over wild-type virus-based methods because the virus is essentially devoid of virulent viral components (Li et al., 2018; Nie et al., 2020). For reliability and feasibility, pseudoviruses must have the same diameter and infection elements as SARS-CoV-2 particles. The pseudovirus with similar morphology to SARS-CoV-2 used in this study is based on the lentivirus packaging system and additionally reconstitutes the spike glycoprotein onto the virus surface (Nie et al., 2020; Ou et al., 2020). Although the pseudovirus showed that the distribution of the spike glycoprotein was not completely consistent with that of SARS-CoV-2, the pseudovirus was safe enough and suitable for this study.

However, whether the pseudovirus also has a response similar to that of wild-type SARS-CoV-2 to changes in environmental conditions needs further confirmation. To confirm this, we detected the attenuation of pseudovirus on the surface of different media and compared it with that reported in previous studies. Previous studies have shown that wild-type SARS-CoV-2 virions exhibit longer viability on stainless steel and PE plastic than on copper or cardboard (van Doremalen et al., 2020). Moreover, with the persistence of SARS-CoV-2 on different surfaces, such as plastic and stainless steel, this virus is viable on these surfaces for several days (Kampf et al., 2020; Kwon et al., 2021). SARS-CoV-2 decays more rapidly on porous surfaces than on nonporous surfaces, but there is no obvious difference in the decay rate of SARS-CoV-2 among nonporous surface types such as PE plastic, stainless steel, or nitrile gloves (Biryukov et al., 2020; Chin et al., 2020). The lower survival ability of pseudoviruses on the surface of porous media than on nonporous media is similar to the results of previous studies (Bhardwaj and Agrawal, 2020; van Doremalen et al., 2020). The decay rate of SARS-CoV-2 pseudovirus on porous surfaces was faster than that on nonporous surfaces (**Figure 1D**), and the mean decay rate of SARS-CoV-2 pseudovirus was not obviously different on nonporous surfaces (**Figure 1E**). It is worth noting that the initial amount of virus has a significant impact on survival time. When the initial amount of virus is 1.78x10^5^ TCID50/ml, SARS-CoV-2 can survive on the stainless steel surface for 3 days, while when the initial amount of virus is 3.38x10^7^ TCID50/ml, SARS-CoV-2 can be detected on the stainless steel surface after 28 days (Geng and Wang, 2023). In this study, a virus starting amount of 6.0 ± 0.2 log TU/mL was used, which comprehensively considered the virus amount in the study from Pan et a (Pan et al., 2020). Due to capillarity, the mass loss of the liquid drop on the surface of the porous medium and the rapid evaporation of the thin liquid film lead to the faster decay of the coronavirus on the porous medium(Chatterjee et al., 2021). These results show that our SARS-CoV-2 pseudovirus and wild-type virus have similar attenuation trends on the surfaces of different media.

Researchers have reported temperature as an important factor affecting the stability and infectivity of SARS-CoV-2(Chin et al., 2020). First, environmental conditions, such as temperature, have been shown to influence the decay rate of infectious viruses(Aboubakr et al., 2021). SARS-CoV-2 is sensitive to heat but can survive for more than 14 days at 4°C (Chin et al., 2020). Ma et al. showed that viruses contaminating paper surfaces could maintain infectivity for at least 17~24 days at -25°C (Ma et al., 2021b). Reports have suggested that cold-chain conditions could maintain SARS-CoV-2 infectivity and stability for days to weeks (Li et al., 2021). All these findings suggest that maintained infectiousness (days to perhaps weeks) of SARS-CoV-2 under a cold-chain environment could be associated with virus stability. We found that our pseudoviruses are similar to wild-type viruses and can survive longer under low-temperature conditions. Cold-chain temperature prolonged SARS-CoV-2 pseudovirus survival for at least 20 days (**Figure 2A**) and decreased the decay rate of the virus compared to that with *in vitro* exposure at room temperature (**Figure 2B**). Similar to the research results of Sun et al (Sun et al., 2022), our experimental results found that the survival time of SARS-CoV-2 in frozen seawater was more than 8 days, but our research also compared the influence of seawater and deionized water on the activity of SARS-CoV-2 under freezing and thawing conditions. In summary, the characteristics of SARS-CoV-2 pseudovirus were consistent with those of wild-type SARS-CoV-2. The above results show that our pseudovirus is similar to SARS-CoV-2 in their responses to changes in environmental factors, providing a virus model for subsequent studies of virus stability under different influencing factors in the cold-chain environment.

There have been many studies on the surface viability of SARS-CoV-2 in various meats, fish, and vegetables (Feng et al., 2021; Bailey et al., 2022), which suggest that COVID-19 may indeed be transmitted through frozen food. Bailey et al. have shown that when SARS-CoV-2 pseudovirus is placed on the surface of chicken, salmon, or pork and stored at 4°C (cold-storage standard temperature) and -20°C (cold-storage standard temperature) for 20 days, the infectivity of the virus only slightly decreases (Bailey et al., 2022). However, we are more concerned about the risk of transmission of COVID-19 in cold-chain transportation. Cold-chain transportation staff are one step earlier than the food sales staff and consumers in terms of contact with goods that may contain live SARS-CoV-2. In addition, staff with COVID-19 may leave SARS-CoV-2 on the surface of goods (Li et al., 2021). At present, there are few surveys on the spread of COVID-19 in cold-chain transportation, including data surveys, virus transmission rules, and the impact of the cold-chain environment on virus vitality, which need to be further supplemented. It is necessary to explore the attenuation of viruses in different environmental conditions during cold-chain transportation.

In the cold chain, PE plastic or cartons with thermal insulation layers are commonly used packaging materials. In addition, during the transportation of frozen food to different places, it is inevitable that the items will be exposed to different temperatures, accompanied by the freezing and thawing of the liquid on the surface of the packaging. We simulated freezing and thawing in the *cold chain* transportation process. The results of freeze−thaw experiments suggest that salt ions in seawater can slow the decay rate of SARS-CoV-2 pseudovirus, but the specific reasons need to be further studied. Researchers have shown that salt enhances the thermostability of viruses by increasing van der Waals forces(Meister et al., 2020). Although SARS-CoV-2, with a lipid envelope, is more tolerant to low temperatures than viruses without an envelope (Yao et al., 2020; Isachenko et al., 2021), it still can freeze. Without cryoprotectants, the protein activity will be affected by the freezing and thawing rate, and the structural protein on the virus envelope may change due to freezing and thawing (Cao et al., 2003). However, at present, the main purpose of most studies is to preserve the activity of viruses or proteins in cryopreservation by using a cryobuffer, such as solutions containing sugar, glycerine, phosphate buffer, etc. (Pikal-Cleland et al., 2000; Arakawa et al., 2001; Bee et al., 2022).There are few studies on the effect of seawater on the survival of viruses. Sun et al. put SARS-CoV-2 in artificial seawater and found that at 4°C, the lower titer of the virus can survive for 7 days, while the higher titer can survive for more than 14 days (Sun et al., 2022). And there is no relevant report on the effect of seawater freezing and thawing on the survival of SARS-CoV-2. When seawater remains on the packaging surface of frozen goods, the low freezing point of seawater can effectively slow the freezing and thawing speed of liquid during repeated freezing and thawing. During rapid freezing, small ice crystals and a relatively large ice-liquid interface surface area are formed, which increases the exposure of protein molecules to the ice-liquid surface, thus increasing protein damage (Cao et al., 2003). During our experiment, it was observed that the freezing time of the seawater group was significantly longer than that of the deionized water group. We preliminarily speculate that the low freezing point of seawater slows the rate of freezing and thawing of the virus and effectively maintains the structural integrity of the virus. The above research results suggest that when transporting seafood and other goods that may contain seawater, the outer packaging of the goods should be properly washed in advance to reduce the presence of salt ions, which may reduce the risk of virus transmission from food sources.

The residue of commonly used disinfectants will have a certain impact on food or people (Choi et al., 2018; Wu et al., 2022), and it is difficult to ensure safety when applied to cold-chain disinfection. Researchers have indicated that UV light, sunlight, 405 nm visible light, and 400 nm-to-420 nm standard LED light effectively inactivate SARS-CoV-2 at 20°C (Heilingloh et al., 2020; Ratnesar-Shumate et al., 2020; Rathnasinghe et al., 2021). However, sunlight irradiation is inconvenient to apply in the closed cold-chain transportation environment. Although low-intensity irradiation, such as 4 μW/cm^2^ UV-LED irradiation, can achieve bactericidal effects (Kim et al., 2016), long-term UV light treatment affects the flavour and texture of dried fish fillets and fresh meat (Hood, 1980; Park et al., 2014). Several studies have shown that the visible spectrum can inactivate pathogenic microorganisms. For example, LED devices emitting visible light in the range of 400-420 nm, 430-460 nm, or 500-780 nm can inactivate a variety of bacteria and effectively inactivate influenza virus (Maclean et al., 2008). Gardner et al. have found that irradiation with a wavelength of 405 nm of 16.8 mW/cm^2^-20.8 mW/cm^2^ has an inactivation effect on the feline infectious peritonitis virus (FIPV) (Gardner et al., 2021). Considering that strong light intensity may affect the quality of food (Glowacz et al., 2015), we used LED visible light with wavelengths of 450 nm to 780 nm and a light irradiance of 4 mW/cm^2^ for the inactivation light source, which effectively decontaminated and degraded the genomic RNA of SARS-CoV-2 pseudovirus on the PE package surface in a frozen environment (**Figure 4**). Our findings support that illumination with LED visible light exposure may be an ideal decontamination method for the outside of packages in closed cold-chain transportation. The most suitable lighting conditions in the real-life environment need to be further evaluated.

**Figure 6:**
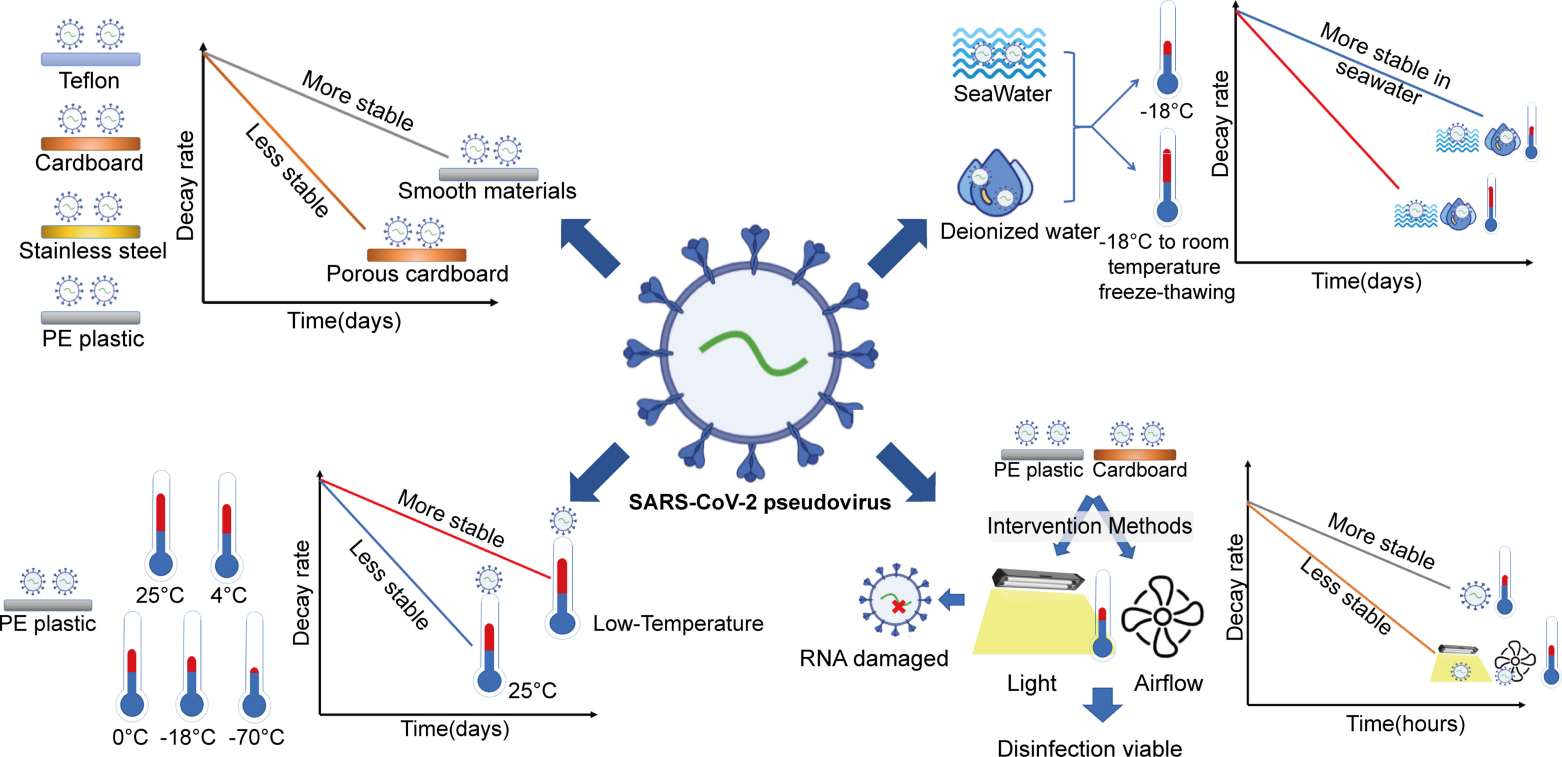
Stability of SARS-CoV-2 in cold-chain transportation environments and the efficacy of disinfection measures. Cold-chain temperature and salt were risk factors that could prolong SARS-CoV-2 viability. Laboratory studies suggested that LED visible light exposure and airflow movement could promote the inactivation of SARS-CoV-2 in the cold-chain environment.

In addition, reports have indicated that an increased air exchange rate and airflow by window opening, air conditioner use, or fan use significantly reduce the infection risk of COVID-19 in cars and on trains at room temperature (Kumar et al., 2021; Shinohara et al., 2021). The airflow environment accelerates the evaporation of droplets, changes the envelope protein structure, and thus affects virus stability at room temperature (Dbouk and Drikakis, 2020; Jayaweera et al., 2020). However, no previous study has evaluated the effect of airflow on SARS-CoV-2 viability in a low-temperature environment. This study indicated that airflow reduced the survival of SARS-CoV-2 pseudovirus on the surface of cold-chain cargo materials. However, some viruses may be blown off the surface in the form of aerosols. Similar phenomena exist in real life. In cold-chain transportation, engineers can install filter membranes at the air inlet and outlet of air conditioning pipes, which reduces the risk of aerosol transmission while reducing the survival of viruses on the surface of goods. Although the light and airflow conditions in this study are relatively simple and limited, they also have an obvious inactivation effect on SARS-CoV-2 (**Figure 6**). This research innovatively explored the viability of COVID-19 and intervention measures in the cold chain cold state, which is a meaningful starting point for research and provides a reference for future response to the new COVID-19, which needs further research. Further study of suitable disinfection conditions is planned based on the actual situation.

## 5 Conclusions

In this study, a nonpathogenic SARS-CoV-2 pseudovirus model was established to investigate the object-to-human transmission route of COVID-19 and to explore effective disinfection methods in a cold-chain environment. The research showed that cold-chain temperature and salt were risk factors that could prolong SARS-CoV-2 viability and increase the risk of SARS-CoV-2 transmission. Laboratory studies suggested that LED visible light exposure and airflow movement could promote the inactivation of SARS-CoV-2 in the cold-chain environment.

## Data availability statement

The raw data supporting the conclusions of this article will be made available by the authors, without undue reservation.

## Author contributions

JD, and XB administered the study and helped with funding acquisition. SP, GL, XG, YL, HX, WQ, HZ, JZ, BL, WS, XH, and GZ conduct the experiment and initial data analysis. SP wrote the manuscript. JP and YL revised and proofread the manuscript. All authors contributed to the article and approved the submitted version.
