# Supplementary material for: Stability of SARS-CoV-2 in cold-chain transportation environments and the efficacy of disinfection measures
Source: Front Cell Infect Microbiol. 2023 Apr 19;13:1170505. doi: 10.3389/fcimb.2023.1170505 (PMC10154586; doi:10.3389/fcimb.2023.1170505)
Supplement: Supplementary file 1 [file Table_1.docx]

**Supplementary materials-Tables**

| **Table S1**. The decay rate of SARS-CoV-2 pseudovirus on different container surfaces | | | |
| --- | --- | --- | --- |
| Surfaces | *k* (mean ± SD) | [95% CI slope] | R2 |
| Teflon film | 0.715±0.118 | -0.957 to -0.474 | 0.76 |
| Cardboard | 1.037±0.213 | -1.50 to -0.576 | 0.65 |
| Stainless steel | 0.634±0.166 | -0.992 to -0.275 | 0.53 |
| PE Plastic | 0.670±0.132 | -0.955 to -0.386 | 0.67 |
| NS: Non-significant deviation from the model; SD: standard deviation. | | | |

| **Table S2.** The decay rate of SARS-CoV-2 pseudovirus on cold-chain temperature conditions | | | |
| --- | --- | --- | --- |
| Temperature | *k* (mean ± SD) | [95% CI slope] | R^2^ |
| 25℃ | 0.872±0.089 | -1.071 to -0.674 | 0.91 |
| 4℃ | 0.097±0.030 | -0.164 to -0.029 | 0.51 |
| 0℃ | 0.012±0.030 | -0.176 to -0.056 | 0.65 |
| -18℃ | 0.090±0.028 | -0.154 to -0.026 | 0.5 |
| -70℃ | 0.080±0.022 | -0.129 to -0.030 | 0.57 |
| NS: Non-significant deviation from the model; SD: standard deviation. | | | |

| **Table S3.** The decay rate of SARS-CoV-2 pseudovirus under seawater and deionized water at-18℃ | | | |
| --- | --- | --- | --- |
| Temperature | *k* (mean ± SD) | [95% CI slope] | R2 |
| -18℃ - Seawater | 0.141±0.009 | -0.1595 to -0.1224 | 0.95 |
| -18℃ - Deionized water | 0.184±0.020 | -0.2272 to -0.1402 | 0.87 |
| freeze-thawing - Seawater | 0.133±0.011 | -0.1573 to -0.1093 | 0.97 |
| freeze-thawing-Deionized water | 0.2538±0.013 | -0.2813 to -0.2263 | 0.92 |
| NS: Non-significant deviation from the model; SD: standard deviation. | | | |

| **Table S4.** The decay rate of SARS-CoV-2 pseudovirus under the visible light illumination | | | |
| --- | --- | --- | --- |
| Groups | *k* (mean ± SD) | [95% CI slope] | R2 |
| PE plastic-Dark | 0.002±0.003 | -0.003 to -0.002 | 0.8684 |
| PE plastic-Light | 0.004±0.005 | -0.005to -0.002 | 0.6713 |
| Cardboard-Dark | 0.003±0.001 | -0.004 to -0.002 | 0.8305 |
| Cardboard-Light | 0.007±0.001 | -0.010 to -0.004 | 0.7587 |
| NS: Non-significant deviation from the model; SD: standard deviation | | | |

**Table S5.** The decay rate of SARS-CoV-2 pseudovirus under the air circulation

| Groups | *K* (mean ± SD) | [95% CI slope] | R^2^ |
| --- | --- | --- | --- |
| PE plastic - calm | 0.045±0.005 | -0.056 to -0.035 | 0.87 |
| PE plastic - airflow | 0.064±0.008 | -0.082 to -0.046 | 0.82 |
| Cardboard - calm | 0.051±0.006 | -0.063 to -0.039 | 0.87 |
| Cardboard - airflow | 0.075±0.011 | -0.099 to -0.051 | 0.80 |

NS: Non-significant deviation from the model; SD: standard deviation
